# Measurement of tumour size with mammography, sonography and magnetic resonance imaging as compared to histological tumour size in primary breast cancer

**DOI:** 10.1186/1471-2407-13-328

**Published:** 2013-07-05

**Authors:** Ines V Gruber, Miriam Rueckert, Karl O Kagan, Annette Staebler, Katja C Siegmann, Andreas Hartkopf, Diethelm Wallwiener, Markus Hahn

**Affiliations:** 1 Department of Obstetrics and Gynecology, University Hospital of Tuebingen, Calwer Street 7, 72076, Tuebingen, Germany; 2 Institute of Pathology and Neuropathology, University Hospital of Tuebingen, Liebermeister Street 8, 72079, Tuebingen, Germany; 3 Department of Radiology, Diagnostic and Interventional Radiology, University Hospital of Tuebingen, Hoppe-Sailer-Street 3, 72076, Tuebingen, Germany

**Keywords:** Breast cancer, Tumour size, Sonography, Mammography, Magnetic resonance imaging, Ductal carcinoma in situ, Invasive ductal carcinoma, Invasive lobular carcinoma

## Abstract

**Background:**

Tumour size in breast cancer influences therapeutic decisions. The purpose of this study was to evaluate sizing of primary breast cancer using mammography, sonography and magnetic resonance imaging (MRI) and thereby establish which imaging method most accurately corresponds with the size of the histological result.

**Methods:**

Data from 121 patients with primary breast cancer were analysed in a retrospective study. The results were divided into the groups “ductal carcinoma in situ (DCIS)”, invasive ductal carcinoma (IDC) + ductal carcinoma in situ (DCIS)”, “invasive ductal carcinoma (IDC)”, “invasive lobular carcinoma (ILC)” and “other tumours” (tubular, medullary, mucinous and papillary breast cancer). The largest tumour diameter was chosen as the sizing reference in each case. Bland-Altman analysis was used to determine to what extent the imaging tumour size correlated with the histopathological tumour sizes.

**Results:**

Tumour size was found to be significantly underestimated with sonography, especially for the tumour groups IDC + DCIS, IDC and ILC. The greatest difference between sonographic sizing and actual histological tumour size was found with invasive lobular breast cancer. There was no significant difference between mammographic and histological sizing. MRI overestimated non-significantly the tumour size and is superior to the other imaging techniques in sizing of IDC + DCIS and ILC.

**Conclusions:**

The histological subtype should be included in imaging interpretation for planning surgery in order to estimate the histological tumour size as accurately as possible.

## Background

The key importance of imaging methods in breast diagnostics lies in the detection and sizing of areas suspicious for malignancy. Breast results are classified using the BI-RADS (Breast Imaging Reporting and Data System) classification [1,2].

Exact pre-therapeutic tumour sizing using imaging methods plays a central role. For example, the possibility of breast conserving treatment significantly depends on the relationship between the tumour-to-breast size. Furthermore, the indication for primary systemic treatment is made, amongst other things, from the tumour size.

The aim of this study was to analyse which of the given imaging methods is the most accurate in the pre-therapeutic sizing of primary breast cancer.

## Methods

121 patients with primary breast cancer who presented preoperatively in the University Breast Centre of Tuebingen between June 2005 and July 2007 were retrospectively analysed. All patients fulfilled the following criteria: primary breast cancer, no neoadjuvant chemotherapy, complete documentation of the tumour size from mammography, sonography and magnetic resonance imaging (MRI) as well as the postoperative histological tumour size. The largest tumour diameter was chosen as the sizing reference in each case.

### Diagnostic imaging equipment

Mammography was performed using two digital full field instruments (Senographe 2000 D, GE Healthcare, Munich, Germany; Selenia, Hologic GmbH, Frankfurt am Main, Germany).

Sonography was performed using a linear transducer with a 50 mm width and a frequency of 12 MHz. Models iU22 and HD 11 from Philips (Philips GmbH Healthcare, Hamburg, Germany) were used. The examination was carried out using “Spatial Compound Imaging” and “XRES Adaptive Image Processing”. Measurement of tumor size took the echopoor centre of the lesion and the echogenic halo into account. The clinical examiner held a minimum of the Degum Level 2 qualification.

Magnetic resonance imaging (MRI) was performed using a 1.5 Telsa system (Gyroscan Achieva, Philips GmbH Healthcare, Hamburg, Germany). A T1-weighted dynamic gradient echo sequence (T1w-FFE = Fast Field Echo) with a native and 7 post-contrast medium series was used. Automated bolus injection of 0.16 mmmol Gadobutrol per kg body weight followed by 10 ml saline were infused intravenously. Image post-processing included the generation of subtraction series and reconstruction of a MIP (maximum intensity projection). Imaging analysis was carried out using the digital data with the help of a suitable workstation (View Forum, Philips Healthcare, Hamburg, Germany).

Only physicians who were specialized on breast diagnostics performed and reviewed each imaging.

### Statististical evaluation

The histological results were divided into the groups “ductal carcinoma in situ (DCIS)”, “invasive ductal carcinoma (IDC) + ductal carcinoma in situ (DCIS)”, “invasive ductal carcinoma (IDC)”, “invasive lobular carcinoma (ILC)“ and “other tumours” (tubular, medullary, mucinous und papillary breast cancer). The largest tumour diameter was chosen as the sizing reference in each case. Bland-Altman analysis was used to determine to what extent the imaging tumour size correlated with the histopathological tumour sizes.The mean difference between the imaging and the histological results was calculated and related to the interval in which 95% of the calculated differences were found (LOA = limits of agreement). Size variation on imaging versus pathology were reported as median and interquartile range, using Box plots. The statistical analysis was performed using SPSS® for Windows (Version 15.0; IBM, Chicago). The level of significance was defined as a p-value of <0.05.

There was no objection against the study from the local ethic committee.

## Results

### Patient collective and malignancy assessment using imaging

121 patients with primary breast cancer were evaluated in a retrospective analysis. The median age was 57 years (range 35–92). An IDC was present in 33.9% of the cases. 31.4% of the patients were allocated to the IDC + DCIS tumour group, and a DCIS alone or ILC alone were found in 12.4% and 14.9% respectively. “Other tumours” occurred in 7.4% of the cases.

The density level of the breast tissue was graded using mammography according to the American College of Radiology (ACR) [1] classification system. In doing so, 8.3% exhibited predominantly lipomatous glandular tissue (ACR I). A mammographic density grade II was present in 29.8% and a density grade III in 47.9%. 13.2% of women had very dense glandular tissue (ACR IV). Density grading was not carried out in one case (0.8%). Malignancy assessment using imaging was performed according to the BI-RADS classification system [1], whereby 96.6% of the sonographic results, 90.9% of the mammography results and 100% of the MRI results were pre-interventionally classified as BI-RADS 4 or higher (Table 1).

**Table 1:** Correlation between the BI-RADS classification (Breast Imaging Reporting and Data System) and the histology for the corresponding imaging method

	**BI-RADS classification**	**Isolated DCIS**	**IDC-DCIS**	**Isolated-IDC**	**Isolated ILC**	**Other tumours**	**Total**
**Diagnostic imaging**		**N (%)**	**N (%)**	**N (%)**	**N (%)**	**N (%)**	**N (%)**
*Sonography*	**0**	0 (0%)	0 (0%)	0 (0%)	0 (0%)	0 (0%)	**0 (0%)**
	**1**	0 (0%)	0 (0%)	0 (0%)	0 (0%)	0 (0%)	**0 (0%)**
	**2**	0 (0%)	0 (0%)	0 (%)	0 (0%)	1 (11.1%)	**1 (0.8%)**
	**3**	0 (0%)	0 (0%)	3 (7.3%)	0 (0%)	0 (0%)	**3 (2.5%)**
	**4**	10 (66.7%)	17 (44.8%)	10 (24.4%)	8 (44.4%)	5 (55.6%)	**50 (41.3%)**
	**5**	5 (33.3%)	20 (52.6%)	28 (68.3%)	10 (55.6%)	3 (33.3%)	**66 (54.5%)**
	**6**	0 (0%)	1 (2.6%)	0 (0%)	0 (0%)	0 (0%)	**1 (0.8%)**
*Mammography*	**0**	0 (0%)	1 (2.6%)	0 (0%)	1 (5.6%)	0 (0%)	**2 (1.7%)**
	**1**	0 (0%)	0 (0%)	0 (0%)	0 (0%)	0 (0%)	**0 (0%)**
	**2**	0 (0%)	0 (0%)	0 (0%)	0 (0%)	0 (0%)	**0 (0%)**
	**3**	1 (6.7%)	4 (10.5%)	3 (7.3%)	0 (0%)	1 (11.1%)	**9 (7.4%)**
	**4**	1 (6.7%)	12 (31.6%)	17 (41.5%)	11 (61.1%)	5 (55.6%)	**46 (38.0%)**
	**5**	13 (86.6%)	19 (50%)	17 (41.5)	6 (33.3%)	3 (33.3%)	**58 (47.9%)**
	**6**	0 (0%)	2 (5.3%)	4 (9.7%)	0 (0%)	0 (0%)	**6 (5.0%)**
*MRI*	**0**	0 (0%)	0 (0%)	0 (0%)	0 (0%)	0 (0%)	**0 (0%)**
	**1**	0 (0%)	0 (0%)	0 (0%)	0 (0%)	0 (0%)	**0 (0%)**
	**2**	0 (0%)	0 (0%)	0 (0%)	0 (0%)	0 (0%)	**0 (0%)**
	**3**	0 (0%)	0 (0%)	0 (0%)	0 (%)	0 (0%)	**0 (0%)**
	**4**	0 (0%)	2 (5.3%)	4 (9.8%)	0 (0%)	0 (0%)	**7 (5.8%)**
	**5**	9 (60%)	22 (57.9%)	26 (63.4%)	8 (44.4%)	3 (33.3%)	**68 (56.2%)**
	**6**	6 (40%)	14 (36.8%)	11 (26.8%)	9 (50%)	6 (66.7%)	**46 (38.0%)**

*DCIS* Ductal carcinoma in situ, *IDC* Invasive ductal carcinoma, *ILC* Invasive lobular carcinoma.

### Comparison of histological sizing with the sizing indication from sonography, mammography and MRI

**Figure 1:**
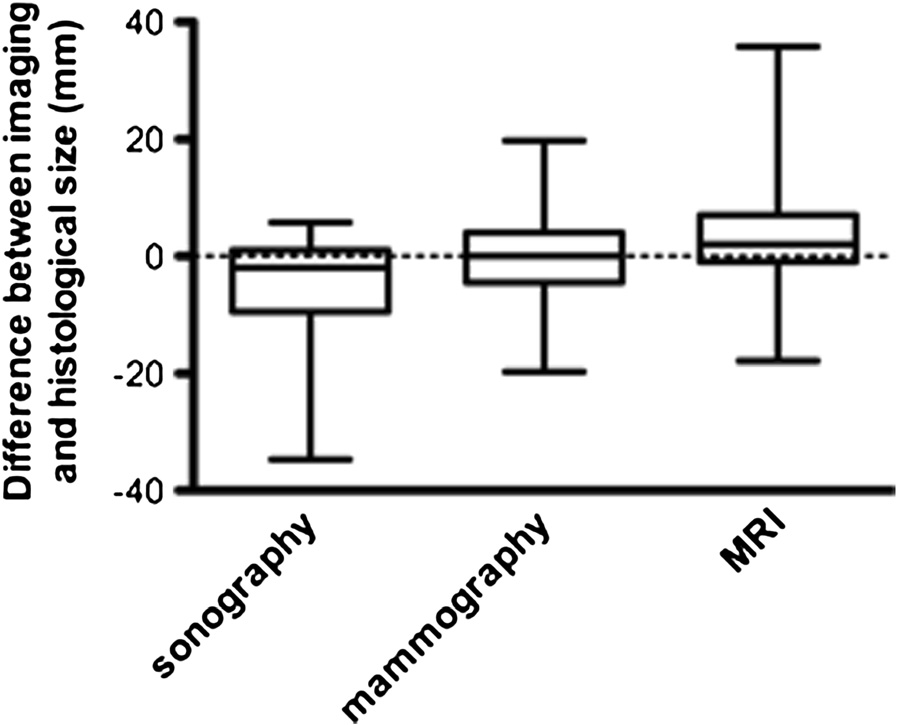
Box Plots illustrating the median size difference between imaging (sonography, mammography and MRI) and histology and the corresponding interquartile range with whiskers from the 5th to the 95th percentile.

**Figure 2:**
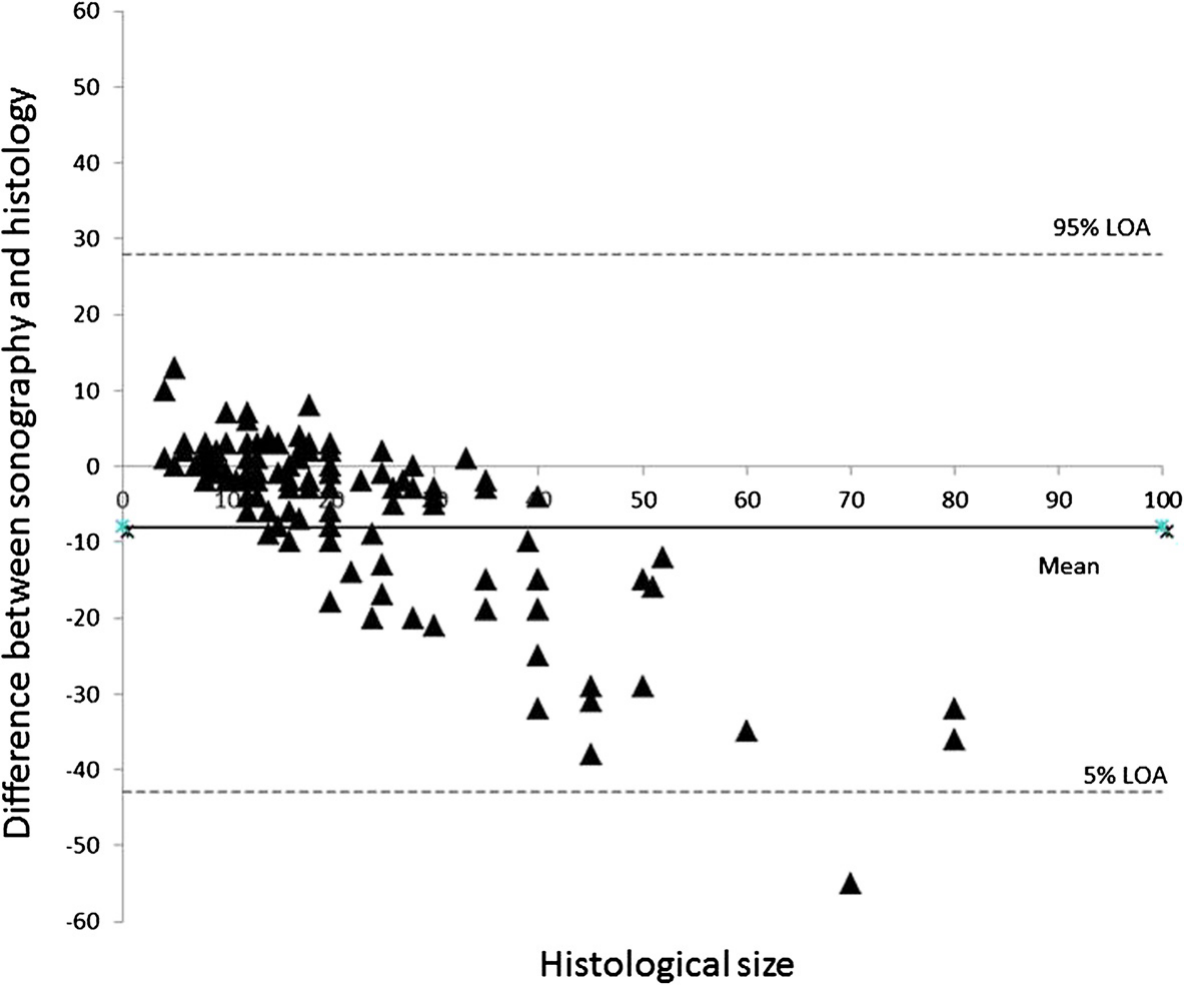
Bland Altman Plots illustrating the size difference between sonography and histology compared to the histological tumour size.

As demonstrated in Table 2, there was a mean difference between the sonographic and histological sizing of -8 mm (LOA: -43 to 28 mm). The median difference was -2 mm (interquartile range: -10 to 1 mm, Figure 1). In the total sonographic collective, there was a highly significant underestimation of tumour size (Table 2), which can be particularly seen in histologically larger lesions (Figure 2). Based on the individual tumour groups, a significant underestimation of size was detected for IDC + DCIS, IDC and ILC (Table 2).

**Table 2:** Comparison of the imaging size and the histological tumour size

**Tumour group**	**Difference between**								
	**Sonography and histology**			**Mammography and histology**			**MRI and histology**		
	**M (mm)**	**LOA (mm)**	**r**	**M (mm)**	**LOA (mm)**	**r**	**M (mm)**	**LOA (mm)**	**r**
DCIS	−15	−87 to 56	0,304	−1	−71 to 68	0,374	5	−46 to 56	0,744
IDC - DCIS	−9*	−47 to 30	0,570	−4	−43 to 35	0,502	2	−46 to 49	0,311
IDC	−4*	−20 to 13	0,853	3	−16 to 22	0,821	3	−19 to 26	0,732
ILC	−10**	−31 to 11	0,853	1	−20 to 13	0,821	2	−31 to 34	0,732
Other tumours	−1	−9 to 6	0,907	3	−8 to 14	0,867	−2	−14 to 10	0,752
TOTAL	−8**	−43 to 28	0,525	−1	−36 to 34	0,550	2	−34 to 39	0,554

Significant differences *p < 0,05 **p < 0,001.

*DCIS* Ductal carcinoma in situ, *IDC* Invasive ductal carcinoma, *ILC* Invasive lobular carcinoma, *M* mean, *LOA* limits of agreement, r - correlation coefficient.

**Figure 3:**
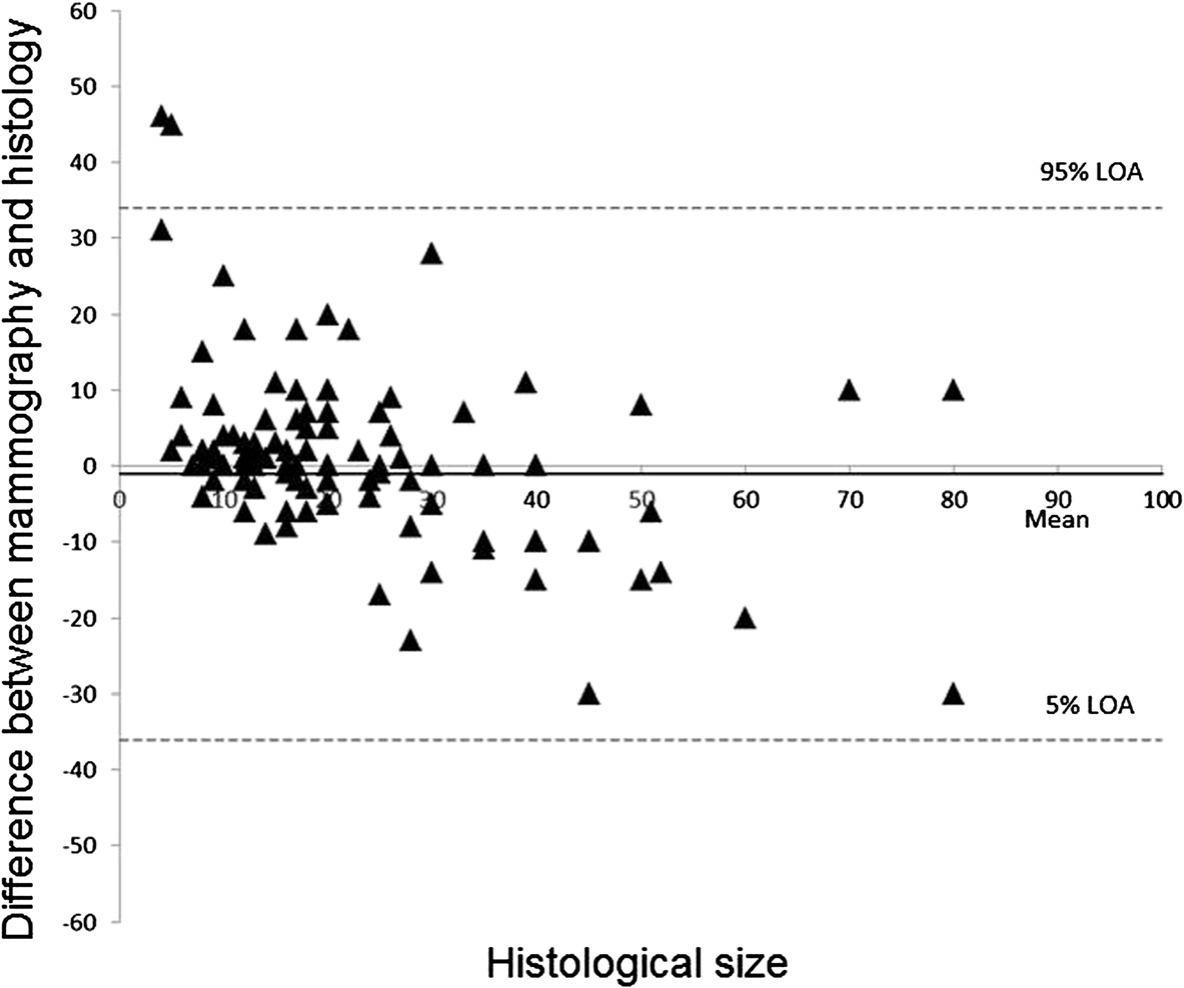
Bland Altman Plots illustrating the size difference between mammography and histology compared to the histological tumour size.

The mean difference between mammography and histology was -1 mm (LOA: -36 to 34 mm, Table 2). The median difference was 0 mm (interquartile range: -5 to 4 mm, Figure 1). There was a non-significant underestimation of size in the whole collective (Figure 3) with no significance based on the individual tumour groups (Table 2).

**Figure 4:**
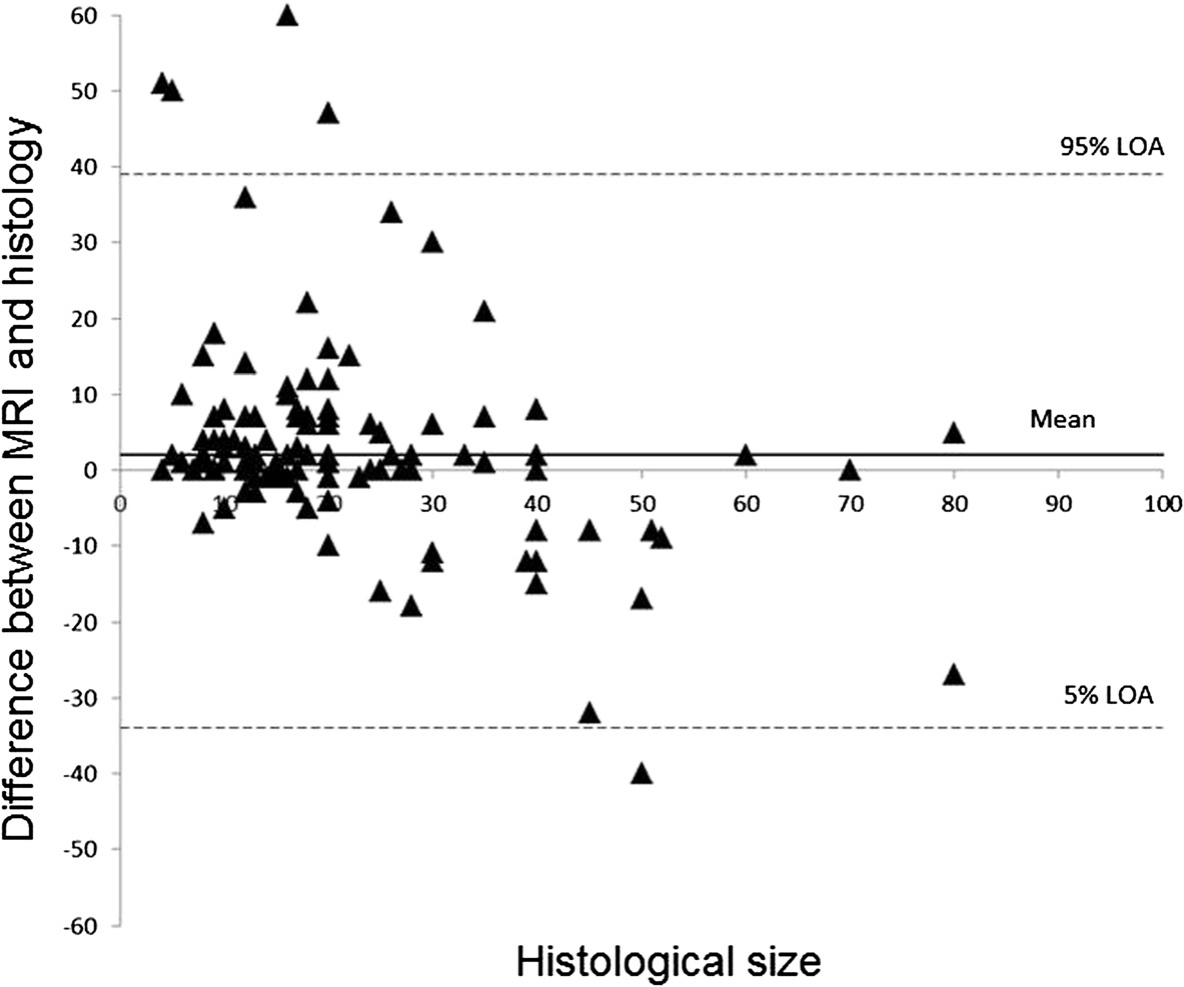
Bland Altman Plots illustrating the size difference between MRI and histology compared to the histological tumour size.

Using MRI, the mean difference in sizing, as compared to the histological tumour size, was found to be 2 mm (LOA: -34 to 39 mm, Table 2). The median difference was 2 mm (interquartile range: -1 to 7 mm, Figure 1), which corresponded with a non-significant size overestimation in the whole collective (Figure 4). No significant correlations were found within the individual tumour groups (Table 2).

## Discussion

### Breast lesion sizing by ultrasound imaging, mammography and MRI in comparison to histopathological sizing

This retrospective analysis showed a highly significant underestimation of the mean histological tumour size with sonography (Table 2; p < 0,001), with a mean of 8 mm. This underestimation increased as the histological result size increased (Figure 2). The investigations by Hieken et al. [3], Shoma et al. [4] and Bosch et al. [5] confirmed the sonographic underestimation of the histological tumour size. Hieken et al. [3] attributed this to the unclear margins of sonographic results from extensive intraductal in-situ components. Bosch et al. [5] linked the underestimation with tumour size, with the image presentation exceeding what is possible with the transducer. An alternative technique here would be the panorama mode. This technique allows a complete image to be built from individual sectional sonographic images. The sonographic measurement of masses whose diameters exceed the width of the transducer is thereby made more accurate.

With regard to mammography, our data also show an underestimation of the mean histological result of 1 mm, although this was not found to be significant (Table 2). The study by Hieken et al. [3] also showed a size underestimation with mammography, which was attributed to the high compression of the breast during the examination. Furthermore, the mammographic size estimation is also negatively affected by breast density.

A non-significant size overestimation of 2 mm in the whole collective was found according to our analysis of the MRI results (Table 2). Onesti et al. [6] described a significant MRI mean overestimation of 1.06 cm, especially for results of >2 cm in size. This can be traced back to tumours with larger DCIS-components or a higher proportion of fibrotic tissue.

Studies which comparatively analysed the diagnostic measurement accuracy of mammography, sonography and MRI came to the conclusion that MRI offered the best correlation with the histological tumour size [7-9]. For a mean histological tumour size of 2.76 cm, Wasif et al. [7] identified a mean tumour site of 2.1 cm using mammography, 1.73 cm using sonography and 2.65 cm with MRI. In a study by Boetes et al. [9] the tumour size with mammography and sonography was underestimated in 14% and 18% of the results respectively, whereas MRI did not show any significant deviation from the histological sizing.

### Significant underestimation of the histological sizing with ultrasound depending on the tumour type

Out data showed a significant underestimation of the histological size with ultrasound with regard to the tumour groups IDC-DCIS (p = 0.008), IDC (p = 0.008) and ILC (p = 0.001). The greatest mean difference between the sonographically measured tumour size and the actual histological tumour size was found for invasive lobular breast cancer (Table 2). Pritt et al. [10] also described the greatest sonographic size underestimation for ILC compared to IDC or ILC-IDC, with a median of 7.5 mm. Our analysis gave a mean size underestimation of 10 mm in this group. Diagnostic demarcation of the tumour using imaging is made more difficult because of the diffuse, infiltrative growth pattern of ILC [11]. Furthermore, ILC tends towards multifocality because of the formation of peritumoral satellite foci, and the additional use of MRI for surgical planning is justifiable, as shown by Rodenko GN et al. [12].

No significant differences between tumour types and histologically established tumour sizes could be found in our study for mammography and for MRI.

### Influencing factors of imaging

In contrast to sonography and MRI, the sensitivity of mammography is significantly negatively affected by increasing breast tissue density [13-15]. Mammographic sensitivity is therefore 30-48% for ACR IV dense glandular breast tissue [13,15] , and mostly breast cancer can only be inadequately displayed with this technique (occult). Breast density also influences the exact sizing of tumours. According to the inclusion criteria definition of our study, results were only included which were visible by all three imaging techniques (mammography, sonography and MRI). Overall, there were no significant variations from the histological tumour size for mammography (Table 2).

If imaging malignancy assessment with reference to the individual tumour groups is considered, isolated DCIS is clearly more commonly classified as BIRADS 5 (Table 1) with mammography (86.8%) than with sonography (33.3%) in our analysis, despite predominantly occurring (66.6%) in ACR III-IV density glandular breast tissue. This is due to the fact that DCIS is accompanied by typical suspicious microcalcification in 73 – 98%, which can be identified mammographically independently of the density of the glandular breast tissue [16-18]. Microcalcification is inadequately seen with ultrasound [19-21]. Soo et al. [21] demonstrated that sonographically conspicious lesions were only detected in 23% of mammographically conspicious microcalcificatons. An exact measurement of the extent of microcalcification is not possible with sonography.

When considering ILC, the detection of clinical findings must be regarded as separate from sizing. In a study by Butler et al. [22], 39% of the mammographically occult ILC and 88% of ILC were diagnosed using ultrasound. In our assessment, ILC was present in 14.9% of all tumours. ILC was diagnosed as BI-RADS 5 in 55% with sonography and as BI-RADS 5 in 33.3% using mammography. Ultrasound therefore appears to be superior to mammography in the detection of ILC, whereas mammography can more accurately determine the size than ultrasound.

Sizing of ILC using sonography reveals a significant underestimation of tumour size compared to mammography (Table 2). In this context, a sonographic influencing factor can be the varying individual interpretation of the malignancy criteria by the various clinicians. For example, the clinical finding size varies depending on whether the hyperechoic margin of a tumour is included or not. In a retrospective analysis, it is always important to question whether all clinicians have interpreted the malignancy criteria in the same way [23,24]. Further malignancy criteria which could result in differing interpretation of the tumour size are the dorsal acoustic attenuation, the blurred margin and as well as infiltration of the vessels in Doppler sonography [25,26]. Although sonoelastography presents with a lower interobserver variability than conventinal B-mode imaging, Isermann et al. [27] found no significant advantage in breast lesion sizing of this technique.

Modern ultrasound equipment also usually operates with complex image processing software. A danger of the image processing is that clinical findings are modified or so embellished that the interpretation of the classical malignancy criteria done up to now is no longer possible [28]. This could also lead to anomalies in the sizing of focal findings.

In contrast to mammography and sonography, all tumours were correctly preoperatively classified as requiring further clarification (> BI-RADS IV) with MRI, (Table 1), and 38% of cases were already histologically confirmed (BI-RADS 6). With regard to sizing, there is a non-significant overestimation of size with MRI in all tumour groups. Analogous to our data, other studies [29-32] show that MRI is superior to both mammography and sonography in the diagnosis of DCIS and ILC. In a study by Kuhl et al. [30], MRI showed sensitivity for all DCIS cases, whether with or without microcalcification, of 98%. For mammography, which relies on the interpretation of suspicious microcalcification and therefore does not detect all DCIS cases, the sensitivity was only 52% [30]. Berg et al. [29] could also show that MRI exhibited a sensitivity of 89% compared to 55% sensitivity for mammography and 47% for ultrasound.

### Study limitations

Investigator influence during the malignancy assessment of the results due to previous knowledge of the results of other imaging techniques cannot be excluded. 38% of the MRI results were BI-RADS 6-lesions and were therefore histologically confirmed first of all. However, this study considered sizing and not malignancy assessment; therefore this does not appear to have any influence on the results. Moreover study population was retrospectively analysed and limited to only those patients with cancer visible on all three imaging modalities.

## Conclusions

According the data from this study (see Table 2), the following points should be observed for the implementation of valid breast cancer sizing:

1. IDC can be measured well with all three imaging methods; MRI and mammography are the more exact methods, whilst sonography showed a significant underestimation of the results.

2. IDC with extensive DCIS involvement can be most accurately measured with MRI. Ultrasound leads to a significant size underestimation on average.

3. According to our data, DCIS alone can be most accurately measured using mammography. Mammography and MRI show no significant variations from the mean tumour size compared to histology.

4. ILC is measured most accurately using MRI and mammography, provided that the results are visible with mammography. Sonography leads to a significant underestimation of the mean tumour size.

From these results, we conclude that for surgical planning, the histological subtype should be included in the imaging interpretation in order to estimate the tumour size as accurately as possible.

## Abbreviations

ACR: American College of Radiology; BI-RADS: Breast Imaging Reporting and Data System; DCIS: Ductal carcinoma in situ; IDC: Invasive ductal carcinoma; ILC: Invasive lobular carcinoma; LOA: Limits of agreement; MIP: Maximum intensity projection.

## Competing interests

The authors declare that they have no competing interests.

## Authors’ contributions

IG carried out imaging and measurements. MR carried out measurements. KK participated in the design of the study and performed the statistical analysis. AS participated in the histological workup. KS carried out imaging and measurements. AH participated in the design of the study and performed the statistical analysis. DW participated in the study design and its coordination. MH participated in the study design and its coordination, imaging, measurements and surgery. All authors read and approved the final manuscript.

## Pre-publication history

The pre-publication history for this paper can be accessed here:

http://www.biomedcentral.com/1471-2407/13/328/prepub
